# Supplementary figures and images for: The p.R66W Variant in RAC3 Causes Severe Fetopathy Through Variant-Specific Mechanisms
Source: Cells. 2024 Dec 9;13(23):2032. doi: 10.3390/cells13232032 (PMC11640247; doi:10.3390/cells13232032)

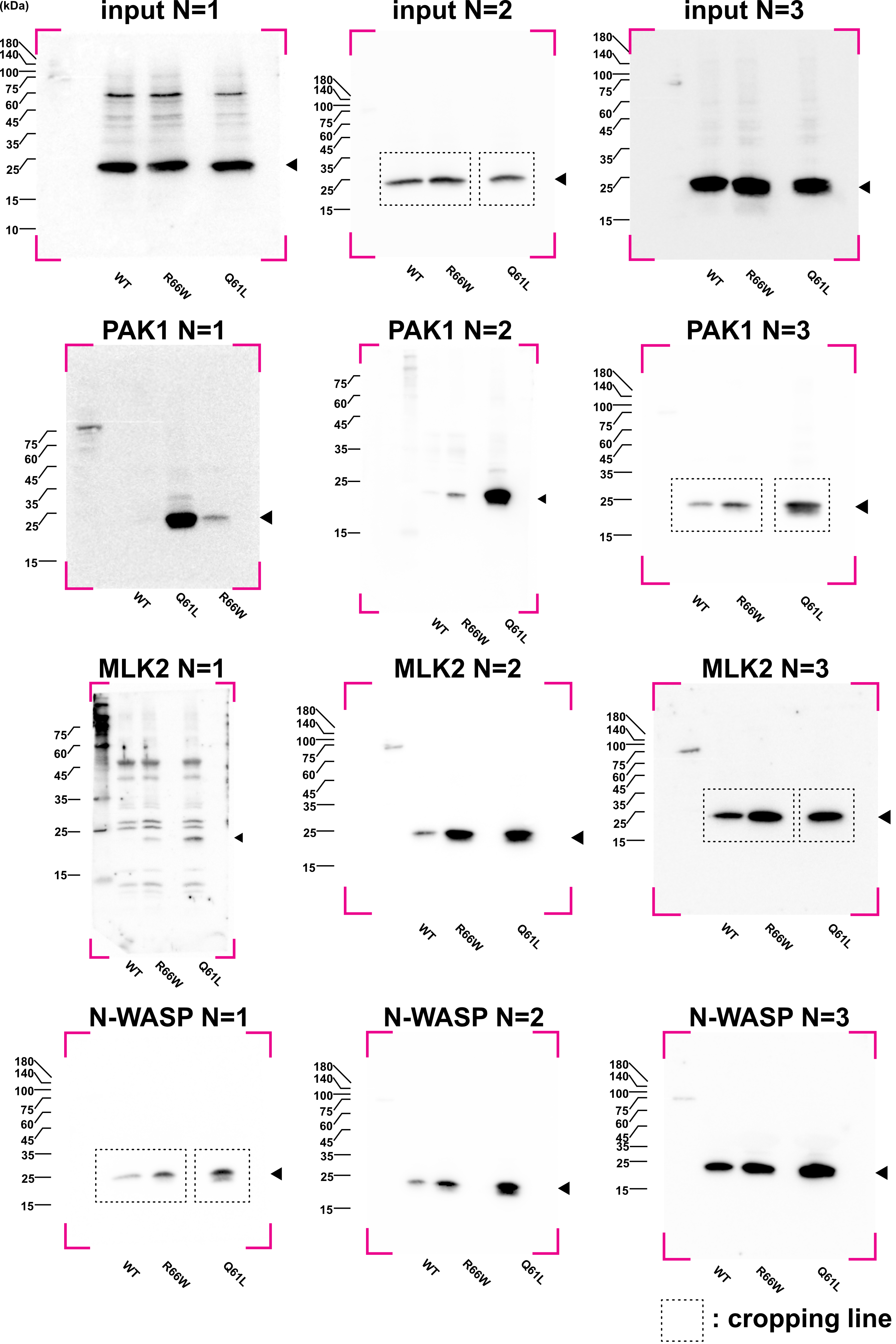

Supplement: Supplementary file 1 [file cells-13-02032-s001.zip › Supplementary Figure S1.tiff]
